# Islamic Sunni Mainstream Opinions on Compensation to Unrelated Live Organ Donors

**DOI:** 10.5041/RMMJ.10046

**Published:** 2011-04-30

**Authors:** Ahmad Natour, Shammai Fishman

**Affiliations:** 1 President, High Muslim Court of Appeal, Jerusalem, Israel, and; 2 Chairman, A. L. Motzkin Association for Promoting Arabic Studies in Israel, Jerusalem, Israel

**Keywords:** Islam, organ donation, transplantation, fatwa

## Abstract

This article focuses on contemporary Islamic attitudes towards the question of compensation to a non-relative live organ donor. This article presents the history of the debate on organ transplantation in Islam since the 1950s and the key ethical questions. It continues by presenting the opinions of the mainstream *ulema* such as Tantawi and Qaradawi. The article ends with a conclusion that there must be no compensation made to a non-related live organ donor, not even a symbolic gift of honor *(ikramiyya).*

Islamic law is a system of jurisprudence that is undergoing processes of renewal and reform in facing the challenges of modern life and science. Mainstream Muslim jurists, known as the *wasatiyya* (centrist) group, are seeking solutions derived from Quranic norms and its spirit. They are looking for solutions that will be in harmony with the higher intentions of *shari’a* and the benefit of the public. Egyptian-born (1926) Sheikh Yusuf Abdullah Qaradawi, considered to be one the most prominent and influential leaders of mainstream-centrist Islam whose views are shared by the Muslim Brotherhood Society, is the author of numerous books and has a religious television show on the al-Jazeera channel.^1^ Sheikh Taha Jabir al-Alwani,^2^ who was born in Iraq in 1935 and spent many years in the United States,^3^ is the founder of the International Institute for Islamic Thought (HIT). These two thinkers, among others, are active in studying the link between Islam and science. They developed the concept of “Islamization of knowledge”, which stands for understanding modern science-based and *shari’a* tools.

The Prophet Muhammad is reported to have said: “There is a cure for every illness, though we may not know it yet.” The search for new treatment methods and applications thereof, if proven successful, is thus strongly recommended. Seeking treatment is not only an individual responsibility but also a collective one. The Prophetic sayings imply that it is the patient’s responsibility to seek out appropriate treatment, the state’s responsibility to establish research institutes, and the scientists’ responsibility to work co-operatively to pursue new means for treatment.^4^ Forming an Islamic approach in topics such as organ donations demands knowledge in medicine as well as in Islamic law. This applies to many questions which are raised in Islam with regard to donations, starting from the matter of brain death which is a core question in the debate on cadaver organ transplantation in Islam. The acceptance by majority opinion of the Council of Islamic Jurisprudence in 1986 of brain death criteria was undoubtedly an influential event.^5^

## THE PROBLEM OF ILLEGAL ORGAN CRIMES IN THE ARAB WORLD

This essay revolves around another factor in the question of donations in Islam, and that is the “sale” of human organs from live donors. In many cases of chronic renal or liver failure and chronic heart diseases, organ transplantation represents the only treatment.^4^ In the countries in the Middle East, like most developing countries, a large proportion of kidney transplants are derived from living related donors. Cadaveric donation had been rudimentary, transplants of other solid organs are rare, and the question of commerce in kidneys has been a frequent topic for discussion. During the 1990s, there were significant changes in all these areas. Today, almost all countries in the Middle East have transplant programs,^5^ but, in the 1980s, patients from the Gulf States bought kidneys at transplantation centers in India. Commercial transplantation takes place in Iraq and Egypt. Iran has a particular system for paying kidney donors.^5^

Much of Euro-American scholarship and commentary on Islam tends to portray Muslims either as automatons mindlessly enacting edicts from religious figures, or to relegate religion to the status of “false consciousness”, epiphenomenal to social and material factors that are more “real”. Hamdy points out that the considerations of Muslim patients with regard to donating kidneys or receiving kidneys from live donors are based on a wide range of cultural, religious, and economic variables.^6^

Organ sale and theft and crimes involving organ transplantation, especially kidneys, exist in many developing countries, including the Arab and Muslim world. As we will see, *ulama* take a clear view on this matter. The issue of organ sale is raised again and again, and generally and unanimously *ulama* forbid dubious organ transactions. This article will deal with one specific aspect, and that is compensation to an unrelated live donor. Of course, this happens in a “non-criminal” setting and with consent, but there is always a danger of wrong-doing in such cases.

## HISTORY OF ISLAMIC DISCOURSE

Scholars mention that the first body parts to be transplanted were skin, bone, teeth, blood, and cornea. The first kidney transplant was performed in 1954, the first liver transplant in 1960, and the first heart transplant in 1967. On December 17, 1986, a landmark medical advance was achieved in England with the first combined heart, lung, and kidney transplant. Since the 1950s, Muslim scholars and jurists have been preoccupied with the subject of transplantation. The increasing number of transplants in the late 1970s and 1980s resulted in an increasing number of Muslim scholars’ responsa on the ethical aspects of the issue. Because organ transplants were not mentioned in the Quran and did not exist during the time of the Prophet, Muslim scholars were forced to draw from general norms and rules in Islam. It is stated that the approach was positive overall, but there were some reservations.^7^

Farhat Moazam (female Muslim researcher of Pakistani origin) reports that there is on-going extensive discourse among Muslim scholars and jurists on how to deal with novel moral dilemmas due to rapid advances in medical science and biotechnology since the early 1980s. Many of these publications are available (in Arabic, Persian, Urdu, and English) with details of the discussions and opinions on the permissibility or not within *shari’a* of medical interventions that include tissue and organ transplantation. These opinions may not always be uniform or unanimous, but all are grounded in the four classical *usul al-fiqh* (roots of jurisprudence or legal methodology), which are Quran, Sunna (sayings, deeds, and approvals of the Prophet Muhammad), Qiyas (the analogy), and Ijma’ (the consensus of the scholars). *Maslaha* (public benefit) and *darura* (necessity) principles were adopted as well, such as “necessity makes lawful that which is prohibited”, “hardship calls for relief, and “where it is inevitable, the lesser of the two harms should be done”. Such concepts, we should note, are heavily used in the *wasatiyya* discourse.

*Ulama* and *fuqaha* (Muslim clerics and jurists) from most major Islamic centers in Saudi Arabia, Egypt, Iraq, and other Muslim countries have generally given *fatawa* (singular: *fatwa,* a response of an authoritative Islamic figure – *alim* or *faqih)* in favor of both live and cadaver renal donations and transplantation, as human life is considered sacred. Many have based their opinions on a Quranic verse (al-Ma’idah, 5:32), stating that saving one life is equivalent to having saved all humanity. Examples of influential organizations that have given positive rulings (while also approving brain death criteria) include the Council of Islamic Fiqh Academy of the Muslim World League (Mecca, 1985), the Organization of Islamic Conference (Jeddah, 1988), and the Islamic Organization for Medical Sciences (Kuwait, 1996).^8^

The issue of organ transplantation has been a matter of debate and dispute among great contemporary scholars from around the globe. It has been discussed in various *fiqh* seminars, and many short and detailed works have been compiled on the subject. While the majority of Indo-Pakistani scholars are of the view that organ transplantation is not permissible, Arab scholars and some scholars of the Indian subcontinent give their permission under certain conditions. No group has given general, unconditional permission for the transplantation of organs.^9^

## MAJOR ETHICAL ISSUES REGARDING ORGAN TRANSPLANTATION IN ISLAM

The key ethical issues which the *ulama* considered with regard to organ transplantation are that the human body is God’s property and that it should be returned in good shape upon death. The Prophet’s wife, Ayisha, said that “Breaking the bone of the dead is equal to breaking the bone of the living”,^10^ i.e. no unjustified or unnecessary harm should be done to the dead body. However, the “lesser injury” of hurting the dead is permitted to prevent the “greater injury” of the suffering living person. Saving one life equals saving the entire humanity; therefore, transplantation as a way to save lives should be permitted.^11^ When donating from a living person, a vital organ, such as a heart, cannot be removed. Only organs like a single kidney can be removed because the donor continues to live and his quality of life is not harmed.

Although the body is viewed as a deposit for lifetime by God and man’s ownership temporary, the opinions of most jurists stress that the necessity of the living party prevails.^12^ This idea exists also in Sheikh Yusuf Qaradawi’s materials.^13^

Sahin Aksoy mentions the Prophet’s tradition *(Hadith)* that states: “God has sent both the disease and cure, and there is a cure for every illness. Therefore be treated but do not treat with haram (in a forbidden way).” He continues: “There is a majority consensus that treatment using haram or mahzurat elements is allowable under certain conditions:

1. “The illness should be of a serious nature.
2. “There should be no alternative treatment which uses only *mubah* (permitted) elements.
3. “The doctors should be strongly (and sincerely) convinced that the treatment in question will be effective against the disease.
4. “Even if the treatment period is prolonged, the amount of the questionable elements used in treatment should not exceed the necessary minimum.”

Aksoy says that it is worthwhile at this point to note one of the most frequently employed maxims among Islamic legal scholars: *al-darurat tubih al-mahzurat* (necessity permits the forbidden).

The reasons of those who do not allow organ transplantation:

1. Humans do not have a right of property on their body and organs.
2. “Breaking a bone of the dead is equal to breaking a bone of a living person.”
3. Organ transplantation if allowed would remove the incentive to search for alternative options by scientific research.

The arguments of those who accept organ transplantation:

1. Islam encourages helping others and saving lives. If anyone saves a life, it would be as if he saved the life of all mankind.
2. God loves those who love their fellow humans and try to mitigate the pain and suffering of others and relieve their sorrows.

There is a consensus with regard to permitting under certain conditions:

1. Single organs, like heart, pancreas, and liver, shall not be donated before death.
2. Donation should not be harmful to donor.
3. Organ transplantation should be the last chance of survival.
4. Transplantation should be an established and effective procedure.
5. The donor should donate his organ of his free will.
6. The recipient should consent to the procedure.
7. There should be no monetary transaction benefit from the procedure.

If the organ is taken from a cadaver:

1. The donor should be dead.
2. The donor should have stated in his lifetime that he wants to donate his organs after death or, at least, should not have indicated any fundamental objection to organ donation.
3. The relatives of the deceased should consent to and accept the removal of the organs. If the dead body is unidentified, permission should be sought from the head of state.^4^

Ilyas (and others) wrote that the permanent Committee for Legal Rulings (Fatawa) in Saudi Arabia concluded the following regarding dissection of dead bodies:

1. Dissection to discover if there is a criminal act causing the death is sanctioned.
2. Dissection to see if there is a contagious disease and to then conclude how to stop its spread is sanctioned.
3. Dissection for educational and training purposes is accepted.

A person has legal authority over his own body, attested by the fact that he can hire himself for work which might be difficult or exhausting. He may also volunteer for war which may expose him to death.

In the case of necessity, certain prohibitions are waived, such as when the life of a person is threatened the prohibition against eating carrion (carcass of a dead animal) or drinking wine is suspended.

He has only forbidden you what has died by itself, blood and pork, and anything that has been consecrated to something besides God. Yet anyone who may be forced to do so, without craving or going too far, will have no offence held against him, for Allah is Forgiving, Merciful.

(Quran 2:173)

Ilyas mentions the decision of the Council of Scholars from all the major Muslim Schools of Law in Great Britain:

It is permissible for a living person to donate part of the body such as the kidneys to save the life of another, provided that the organ donated would not endanger the donor’s life and that it might help the recipient.

Finally, he quotes the Hadith of the Prophet:

Whoever helps a brother in difficulty, God will help him through his difficulties on the Day of Judgment.^14^

## OPINIONS OF THE CONTEMPORARY ULAMA ON THE MATTER OF COMPENSATION TO UNRELATED LIVE DONORS

The topic of this article is Islam’s point of view with regard to paying live donors who are unrelated to the beneficiary of the donation. The most recent *fatawas* and articles of the most important Islamic *ulama* of our time show that the *ulama* are unanimously in agreement against the “sale” of human organs for live donors.

The Islamic Fiqh Academy in Jeddah published the final announcement of a conference on organ donations in Islam held in March 2009 in the presence of (the late) Sheikh al-Azhar Muhammad Sayyid Tantawi (Al-Azhar University is a 1,000-year-old institution for high Islamic studies located in Cairo; the Sheikh of al-Azhar is considered a very senior religious leader for the Sunni Islamic world), stating the prohibition of any person on selling any part of his body.

Donation, however, is permitted between relatives. According to the statement: “The human body is sanctified by Allah who had forbidden turning it into an item for commercial sale, purchase, or exchange. Man must be a reliable guard of his body.”^15^,^16^

Sheikh Zaki Badawi, an important religious leader of Muslims in Britain and an expert on *shari’a* who died in 2006, was the most senior Islamic *alim* in Britain. He published an elaborate learned opinion on the matter, together with a long list of important British Islamic personalities. He stated: “Organ donation must be given freely without reward. Trading in organs is prohibited.”^17^

Sheikh Yusuf Abdulla al-Qaradawi opposes “selling” organs because he does not want human organs to become merchandise to be bargained over. However, he allows live donors to unrelated beneficiaries to receive a gift or a gift of honor *(ikramiyya).*^13^

Qaradawi, Badawi, and Tantawi belong to the centrist group, while some Wahhabi (the religious affiliation of the Saudi regime which is considered as offering a harsh literal interpretation of the law) jurists, such as the late IbnBaz (Abdul Aziz ibn Abdullah ibn Baz, served as the Grand Mufti of Saudi Arabia from 1993 until his death in 1999), are totally against any organ transplantation and oppose any sale of human organs from live people.^18^,^19^ Another source belonging to the Wahhabi movement is the book on organ transplantation in Islam by Dr Amin Muhammad Salam al-Batush. He asks the question: “Is the human body the property of its owner?” His answer is that there is no law, nature, or logic which could allow the sale of human body parts, since Allah sanctified and separated humankind from other beings. He also states that saleable goods are those which are detached from the human being, not those which are connected to him.^20^

Hamdy states that all the *ulama* of al-Azhar and dar al-Ifta’ have permitted organ transplantation under the conditions that there be life-saving benefit to the recipient, no harm to the living donor, and no commercial exchange.

Yet one particularly charismatic and important popular religious figure in Egypt, the late Shaykh ash-Sha’arawi (1911–1998), opposed organ transplantation in all its forms. His position, widely publicized in the late 1980s, was premised on the religious tenet that the human body belongs to God alone. “How can you give a kidney that you yourself do not own?”, he famously asked.

## SOME RESEARCHERS’ OPINIONS AND FINDINGS

Nada Muhammad Na’im al-Daqar, in the conclusions to her book, *Mawt al-Dimaghbaina al-Tibb wal-Islam* (Brain Death between Medicine and Islam), mentions that the “sale of organs should be totally banned out of respect for man. I suggest passing criminal laws which forbid anyone to assist in organ sales or participate in their removal, in order to stop this phenomenon which takes place in some Islamic countries.”^21^

In her book, *Bioethics and Organ Transplantation in Muslim Society, A Study in Culture Ethnography and Religion,* Farhat Moazam describes a hospital in Karachi which encourages the ill to find kidneys among their relatives as opposed to taking kidneys from a paid unrelated donor as is done in other hospitals in Pakistan: “Aware of hospitals that use unrelated, paid donors and convinced that this practice is unethical, staff in the Institute accept only blood-related donors chosen from the extended family”. One more important point that Moazam makes is that, due to the low level of medical services in Third World countries, especially in the area of dialysis, many turn to kidney transplantation as the only resort.^8^

Aksoy’s position in favor of monetary compensation:

Although human organs are not ordinary property, it does not mean that any financial transaction associated with the organ should be forbidden. Islam always allowed exceptions, as it is a natural way of life.

Al-Mahdi, Chairman of the Neurosurgery Department at Ibn Sina Hospital in Kuwait, in writing about kidney transplants, concluded that (as quoted by Aksoy), until we can obtain an adequate supply of organs through voluntary and uncompensated donation, we must countenance the possibility of offering donors “material recompense, on condition that no publicity in this respect is made”. The compensation should be half blood money (money paid to the victim or his family for murder or physical injury), which is 5,000 Kuwaiti Dinars. Aksoy also quotes Muhammad Sayyed Tantawi (1989, then Grand Mufti of Egypt): “Man’s sale of any of his organs is lawfully invalid and prohibited. Such sale is only permissible in the rarest cases, decided by reliable doctors when they deem a patient’s life contingent upon that sale.” Aksoy is against considering brain death as death and allows removing an organ from a cadaver even without consent.^4^

The opinion of Ilyas (and others) is that human organs should be donated and not sold. It is prohibited to receive a price for an organ (based on Badawi 1995).^14^

## CONCLUSIONS

The solution revolves around balancing the benefit on one hand and the extent of damage on the other hand. In the case of taking an organ from the deceased, the balance is between the benefit to the receiver of the donation and the damage caused by desecrating a dead body. In the case of taking an organ from a live person, the balance is between the benefit to the receiver of the donation and the possible damage to the donor. This is the reason for forbidding the donation of a single vital organ, such as a heart, as the damage of removing it is the certain death of the donor.

As contemporary *ulama* permitted live donations and not only cadaver donations, the question of compensation or price became unavoidable. It is difficult to control the material aspect in this peculiar situation in which both sides benefit; the rich man is eager to view the poor man’s body as a pool of replacement parts, while the poor man views his body as the ultimate source of money.

Some could argue that the unclear voice of those who represent the position of the *shari’a* on the matter of compensation does not help prevent organ trade. Egypt, a Muslim state, is considered to be in third place in the world in organ trading. In 2010, after a 15-year wait for approval, the government and the parliament approved the law regulating organ transplantation, with the blessing of al-Azhar. Article 4 states that transferring an organ or part of an organ from one person to another will be done only by way of donation. Article 6 was more specific when it stated that a human organ or part of a human organ should not be dealt with in the manner of selling and buying or receiving any type of exchange, by the donor or by his heirs, from the receiver of the donation or from his family. The physician who performs the transplant is forbidden to perform the operation in such a case.

In Morocco, article 5 of law no. 16–98 forbids the donor from receiving a salary or price in exchange for the organ, except the expenses of the operation.

In Lebanon, law no. 109 (September 19, 1983) imposed the condition that the donation be an unconditional gift.

An organ transplantation bill that had been under study with the senate in Pakistan since 1992 was finally approved on September 5, 2007 as “A Transplantation of Human Organs and Tissues Ordinance 2007” by the Government of Pakistan; many illegal organ donation and transplantation centers were closed down, and many senior doctors involved in the organ trade were arrested and punished.^14^

It would be difficult to suppose that the mere permission given by the *ulama* for donations caused this awful situation of wild trade in organs. However, the permission that some *ulama* gave for giving a gift *(ikramiyya)* is misleading and harmful. No matter what it is called, gift, price, or compensation, it is a wrong principle. These *ulama* should have known that such a loop-hole could be taken advantage of, to permit a thriving industry in organ trade. The one and only benefit that a Muslim donor should expect for his donation is a heavenly reward for his *ithar* (altruism). Assuming that *fatawa* have an influence on the conduct of the public, the loop-hole of the *ikramiyya* may cause disorder and allow organ “sales”. If, however, we assume that Muslims do not adhere to *fatawa,* then our conclusion is that the religious prohibition expressed by the *fatawa* in their *fatawa* is not strong enough to resist the financial need of the poor. In this case, penal and social state legislations should be put into action.
